# β-Cryptoxanthin Reduces Body Fat and Increases Oxidative Stress Response in *Caenorhabditis elegans* Model

**DOI:** 10.3390/nu11020232

**Published:** 2019-01-22

**Authors:** Silvia Llopis, María Jesús Rodrigo, Nuria González, Salvador Genovés, Lorenzo Zacarías, Daniel Ramón, Patricia Martorell

**Affiliations:** 1 Cell Biology Laboratory, Food Biotechnology Department, Biópolis SL/Archer Daniels Midland, C/Catedrático Agustín Escardino Benlloch 9, Paterna, 46890 Valencia, Spain; silvia.llopis@adm.com (S.L.); nuria.gonzalez@adm.com (N.G.); Salvador.Genoves@adm.com (S.G.); Daniel.RamonVidal@adm.com (D.R.); 2 Food Biotechnology Department, Instituto de Agroquímica y Tecnología de Alimentos (IATA), Consejo Superior de Investigaciones Científicas (CSIC), C/Catedrático Agustín Escardino 7, Paterna, 46890 Valencia, Spain; mjrodrigo@iata.csic.es (M.J.R.); lzacarias@iata.csic.es (L.Z.)

**Keywords:** β-Cryptoxanthin, carotenoids, *Caenorhabditis elegans*, fat reduction, oxidative stress, transcriptomic analysis, metabolic syndrome, aging

## Abstract

β-Cryptoxanthin (BCX) is a major dietary pro-vitamin A carotenoid, found mainly in fruits and vegetables. Several studies showed the beneficial effects of BCX on different aspects of human health. In spite of the evidence, the molecular mechanisms of action of BCX need to be further investigated. The *Caenorhabditis elegans* model was used to analyze in vivo the activity of BCX on fat reduction and protection to oxidative stress. Dose-response assays provided evidence of the efficacy of BCX at very low dose (0.025 µg/mL) (*p* < 0.001) on these processes. Moreover, a comparative analysis with other carotenoids, such as lycopene and β-carotene, showed a stronger effect of BCX. Furthermore, a transcriptomic analysis of wild-type nematodes supplemented with BCX revealed upregulation of the energy metabolism, response to stress, and protein homeostasis as the main metabolic targets of this xanthophyll. Collectively, this study provides new in vivo evidence of the potential therapeutic use of BCX in the prevention of diseases related to metabolic syndrome and aging.

## 1. Introduction

Xanthophyll β-cryptoxanthin (BCX)—also known as 3-hydroxy-β-carotene ((3R)-β,β-caroten-3-ol)—is primarily synthetized in higher plants by non-heme di-iron β-carotene hydroxylases adding a hydroxyl group at the C3 position of the β-carotene ring [1,2]. Xanthophyll β-cryptoxanthin is a major dietary pro-vitamin A carotenoid, the most important pro-vitamin A xanthophyll in the diet and commonly found in human plasma [3,4,5]. Moreover, several studies suggest that BCX has a relative high bioavailability from common food sources and conversion to retinol can be comparable to that of β-carotene [6,7]. The main dietary sources of BCX are fruits and vegetables; however, only a small number contain significant amounts of BCX; the most common foods rich in this xanthophyll are: sweet red pepper and hot chili peppers, pumpkins, papaya, persimmons, tangerines and sweet oranges, peaches, and sweet corn [6,8]. Among these foods, the citrus tangerines and sweet oranges are considered the main contributors of BCX to the diet in many countries due to their high consumption both as fresh fruit and juice [6,7,9]. In fact, clinical studies indicated that seasonal intake of citrus fruits in many populations significantly increases plasma levels of BCX and its concentration is considered to be a biomarker of citrus fruit consumption [3,10]. Interestingly, the preferential natural accumulating form of BCX in the flesh of ripen citrus fruit is esterified with lauric, myristic, and palmitic acid, and only a minor proportion—ranging from less than 5% up to 20%—remains in free form [11,12].

Besides the well-established function of BCX as a source of vitamin A [3,13], this xanthophyll may exert other beneficial functions in different processes of human health. Evidence indicates that BCX has as potent in vitro antioxidant capacity and may protect human cells against oxidative damage, leading to the suppression of inflammation, acting also as a scavenger of free radicals and preventing the oxidative damage of biomolecules as lipids, proteins, and nucleic acids [14,15,16]. However, there is scarce information regarding in vivo studies where the antioxidant capacity of BCX has been assessed in targeted tissues at suitable concentration relative to the oxidizing agent [6]. Moreover—related with its antioxidant property—BCX may be also beneficial in the prevention of vascular disease [17,18,19,20]. Additionally, other studies propose that a moderated BCX intake may be helpful in reducing the risk of certain cancers [21,22] and in the prevention of age-related cognitive dysfunction in mouse brain [16]. Furthermore, one of the most remarkable biological functions of BCX is its role in bone health [23], since BCX has been proven to play a role in bone homeostasis by promoting osteoclast formation and inhibiting osteoblast action [24,25].

In the case of obesity-related disorders, a relationship between oral intake of BCX with obesity and metabolic syndrome in humans has been described. Thus, some studies established lower serum BCX levels in obese than in non-obese patients, independently of the diet [26,27]. A previous work indicated that a supplementation of BCX in obese subjects improved serum adipocytokine profiles [28]. Other authors described that a continuous oral intake of BCX reduced visceral adipose tissue, body weight, and abdominal circumference in mildly obese males [29,30]. Moreover, oral administration of BCX also repressed body weight and adipocyte hypertrophy in obese model mice [31]. Indeed, some authors concluded that a diet rich in BCX may prevent the development of metabolic syndrome [28,32,33,34]. However—in spite of these evidences—there are few studies focused on the potential molecular targets of BCX upon obesity and metabolic syndrome. Studies in obese diabetic mice indicate that BCX may act on modulating PPARγ (peroxisome proliferator-activated receptors-gamma type) to reduce adipocyte proliferation and hypertrophy, and its chronic inflammation through the downregulation of genes involved in the cell cycle, chemotaxis, and immune system [31,35]. However—as in many other studies—the mechanism of visceral fat reduction has been mainly addressed in mice upon oral administration of a complex food matrix (powder from pulp of Satsuma mandarin) as the source of BCX, thus the potential activity of other compounds cannot be ruled out [31] and the metabolic targets of BCX need to be further investigated using in vivo models.

*Caenorhabditis elegans* is an excellent model organism to study obesity. Previous studies have described the possible mechanisms and the genes involved in the pathways that regulate fat metabolism in this nematode [36,37,38]. Many of the identified genes involved in lipid metabolism have orthologues in humans, sharing also the same control of homeostasis [39]. Furthermore, fat accumulation in lipid droplets are mainly in gut and hypodermal cells, which enables its detection and quantification by different dying techniques. Although there are different methods available to measure lipids in the nematode, each one has advantages and disadvantages. Nile Red is a dye widely used because offers several advantages that are rapid, sensitive, and suitable for live imaging and screening studies [40]. Moreover, it has been used by many authors to identify evolutionarily conserved fat regulatory genes and small molecules that affect fat metabolism when investigated through a variety of independent methodologies [41]. Thereby, several studies have used the nematode *C. elegans* to evaluate potential obesity therapeutics [42,43,44,45]. This organism has been also used to validate functional properties of several carotenoids such as increase of longevity and antioxidant activity [46,47,48,49,50], being relevant to study the antioxidant effect of astaxanthin stereoisomers [51]. Regarding the carotenoids conversion to retinoids, two carotenoid cleavage dioxygenase have been identified in *C. elegans* with similarity to β-carotene 15,15-oxygenase (BCO1) (central cleavage of β-carotene) and β-carotene 9′,10′-oxygenase (BCO2) [52]. The BCO1 role in the nematode, as in other animals, has been shown to be essential in the transformation of β-carotene to retinal [53]. However, there is no previous report about the effects of BCX on *C. elegans* and the molecular mechanisms targeted by this carotenoid.

In the present study, the potential fat-reducing effect and antioxidant activity of BCX have been investigated using the in vivo model system of *C. elegans*. As a result of treatment, nematodes reduced the body fat content and were more resistant to an acute oxidative stress. Global transcriptional analysis revealed that BCX influences energy expenditure, response to stress, and protein turnover.

## 2. Materials and Methods

### 2.1. Carotenoid Standards and Extracts

Carotenoid β-cryptoxanthin (BCX) (purity ≥ 97%) was supplied by Extrasynthese (Lyon, France) and lycopene (purity ≥ 98%) and β-carotene (purity ≥ 97%) by Sigma-Aldrich (Saint Louis, MO, USA). The carotenoid stock solutions were prepared in chloroform and hexane (HPLC grade, Scharlau, Barcelona, Spain) and purity tested by HPLC-DAD (Waters, Barcelona, Spain) (High Performance Liquid Chromatography-Diode Array Detector) using conditions described in Rodrigo et al. [54]. Aliquots for the experiments were prepared from stocks, completely dried under N2 gas and immediately used. Furthermore, carotenoid extracts rich in BCX were obtained from fresh juice of mature Clementine mandarin (*Citrus clementina*) as described in Rodrigo et al. [54] and individual carotenoid composition was determined by HPLC-DAD immediately before use as indicated elsewhere [54]. Briefly, 2-mL aliquots of fresh juice were weighted in screw-capped polypropylene tubes (50 mL), and 4 mL of methanol were added. The suspension was stirred for 10 min at 4 °C. Tris-HCl (50 mM, pH 7.5) (containing 1 M NaCl) was then added (3 mL) and further stirred for 10 min at 4 °C. Chloroform (5 mL) was added to the mixture, stirred for 5 min at 4 °C and centrifuged at 3000× *g* for 10 min at 4 °C. The hypo-phase was removed, and the aqueous phase re-extracted with chloroform until it was colorless. The pooled chloroform extracts were dried on a rotary evaporator at 40 °C. To test the potential effect of esterified versus free carotenoids extracts on in vivo assays, half of the extracts were saponified in a methanolic solution of KOH (6% *w*/*v*) overnight at room temperature. Free carotenoids from saponified extracts were recovered from the upper phase after adding 3 mL of MilliQ water and 5 mL of solution A (petroleum ether:diethyl ether, 9:1) to the mixture. Repeated re-extractions by adding 3 mL of solution A were carried out until the hypo-phase was colorless. Non-saponified samples were dissolved in 5 mL of solution A and 3 mL of MilliQ water and proceeded as described above for saponified extracts. The extracts were reduced to dryness by rotary evaporation at 40 °C, dissolved with chloroform and methanol, and the extract was transferred to a 1.5-mL vial, dried under N2 and kept at −20 °C until HPLC analysis or in vivo assays were conducted. All operations were carried out on ice under dim light to prevent photodegradation, isomerization, and structural changes of the carotenoids.

For in vivo assays dried aliquots of carotenoids (commercial standards and Clementine juice extracts) were dissolved in chloroform: ethanol solution (ratio 1:15, *v*/*v*).

### 2.2. Quantification of Individual Carotenoids in Extracts

To determine the carotenoid profile and concentration in mandarin juice extracts, aliquots were analyzed by HPLC using a Waters liquid chromatography system equipped with a 600E pump (Waters, Barcelona, Spain) and a model 2998 photodiode array detector (DAD) (Waters, Barcelona, Spain), and Empower software (Waters, Barcelona, Spain). A C30 carotenoid column (250 × 4.6 mm, 5 µm) coupled to a C30 guard column (20 × 4.0 mm, 5 µm) (YMC, Teknokroma, Barcelona, Spain) was used. Samples were prepared for HPLC by dissolving the dried carotenoid extracts in chloroform: methanol: acetone (3:2:1, *v*:*v*:*v*). Ternary gradient elution was used for carotenoid separation. The initial solvent composition consisted of 90% methanol, 5% water, and 5% methyl tert-butyl ether (MTBE). The solvent composition changed in linear fashion to 95% methanol and 5% MTBE at 12 min. During the next 8 min, the solvent composition was changed to 86% methanol and 14% MTBE. After reaching this concentration, the solvent was gradually changed to 75% methanol and 25% MTBE at 30 min. After 20 min, the solvent composition changed linearly, being 50% methanol and 50% MTBE at 50 min and maintained at this proportion until 60 min. The initial conditions were re-established in 5 min and equilibrated for 15 min before the next injection. The flow rate was 1 mL/min, column temperature was set to 25°C, and the injection volume was 20 µL. The PAD was set to scan from 250 to 540 nm, and for each elution a Maxplot chromatogram was obtained, plotting each carotenoid peak at its corresponding maximum absorbance wavelength.

Carotenoids were identified by their retention time, absorption, and fine spectra [54,55]. The carotenoid peaks were integrated at their individual maximal wavelength, and their contents were calculated using the appropriate calibration curves as described elsewhere [54,56]. For the quantification of esterified xanthophylls the calibration curves of the corresponding free carotenoids were used.

### 2.3. C. elegans Strain and Treatments

*Caenorhabditis elegans* strain N2, Bristol (wild-type) was provided by the Caenorhabditis Genetics Center (University of Minnesota, Minneapolis, MN, USA). Nematodes were grown and maintained on nematode growth medium (NGM) at 20 °C using *Escherichia coli* OP50 bacteria as food source.

For the evaluation of BCX, worms were grown on NGM as control diet, or NGM supplemented with different doses of BCX (0.005, 0.01, 0.025, 0.05, and 0.1 µg/mL). The carotenoids lycopene and β-carotene were also tested at 0.025 µg/mL. All carotenoids solutions were prepared as described above and added on the agar plate surface. The appropriate amount of chloroform: ethanol (ratio 1:15) was also added on NGM control medium.

In addition, *C. elegans* body-fat reduction was evaluated with NGM plates supplemented with the carotenoid extract from mandarin juice containing BCX at a final concentration of 0.025 µg/mL.

Finally, the activity on *C. elegans* fat-reduction was evaluated in different food matrices supplemented with BCX. Different volumes of each matrix were added to the NGM plates in order to obtain a final dose of 0.025 µg/mL of BCX: dairy fermented product (250 µL/plate), skimmed milk (250 µL/plate), sugar-free soft drink (with or without caffeine) (100 µL/plate) or orange juice (50 µL/plate).

### 2.4. Bioassimilation Assay in C. elegans

In order to confirm the absorption of BCX by the nematodes during feeding experiments, populations of the *C. elegans* strain N2 Bristol were cultured on NGM or NGM supplemented with BCX (1 µg/mL). At least 40 plates per condition were prepared and worms were recovered with M9 buffer and immediately washed three times to remove *E. coli* OP50 present in the media. Additionally, 2 h of incubation in M9 buffer was performed to facilitate the removal of gut microbiota from the nematodes. The evacuated and washed worm pellets (containing approximately 100 mg per condition) were recovered in 2 mL cryotubes and immediately frozen in liquid nitrogen. The pellets were defrosted on ice, transferred to 1.5-mL tubes with 0.5 mL of acetone (HPLC grade, Scharlab, Barcelona, Spain), and disrupted with micro-pestle. Disrupted pellets were vortexed for 30 s and centrifuged for 3 min at 15,700× *g* at 4 °C. The acetone was recovered, and pellets were re-extracted twice with 2 mL acetone. The pooled acetone extract for each condition was dried under nitrogen stream. The dried residue was dissolved in 250 μL dichloromethane (HPLC grade, Scharlab, Barcelona, Spain), and 250 μL MilliQ water was added and vortexed for 30 s. The organic phases were recovered, and aqueous phase were re-extracted twice with 250 μL of dichloromethane. The pooled organic phases were dried under nitrogen stream and stored at −20 °C until HPLC-PAD analysis. The analytical chromatographic and identification conditions used were the same as indicated in Section 2.2.

### 2.5. Body Fat Reduction Assays in C. elegans

For Nile Red assays, age-synchronized nematodes were cultured in NGM or NGM supplemented with the different doses of commercial carotenoids, carotenoid extract from mandarin juice, or the different food matrices supplemented with commercial BCX. The NGM plates with 6 µg/mL of Orlistat (Sigma–Aldrich, Madrid, Spain) were used as positive control. The nematode fat content was measured by Nile Red staining as previously described by Martorell and coworkers [45]. Nile Red (Sigma-Aldrich, St. Louis, MO, USA) was added on the surface of NGM agar plates, previously seeded with *E. coli* OP50, to a final concentration of 0.05 µg/mL. Worms were incubated at 20 °C and when they reached young adult stage, nematodes were placed in M9 buffer and fluorescence was measured in a spectrofluorometer FP-6200 (Jasco, Easton, MD, USA) with λ excitation 480 nm and λ emission 571 nm. At least two independent experiments were performed with each sample.

Nematode triglyceride (TG) content was measured using Triglyceride Quantification Kit (Biovision, Mountain View, CA, USA). Age-synchronized N2 nematodes were treated with BCX at a dose of 0.025 μg/mL until nematodes reached young adult stage. Then, worms were collected and washed with PBS buffer. After worm settling, supernatant was removed and 400 μL of TG assay buffer was added to worm pellet. Nematodes were sonicated with a digital sonifier (Branson Ultrasonics Corporation, Danbury, CT, USA) using 4 pulses for 30 s at 10% power. Samples were slowly heated twice at 90 °C for 5 min in a thermomixer (Eppendorf, Hamburg, Germany) to solubilize all TGs in the solution. After brief centrifugation, samples were used for the TG assay following the manufacturer’s instructions. Five different biological replicates were included for each condition in four independent experiments. Total protein content was estimated by bicinchoninic acid assay (Pierce^TM^ BCA Protein Assay Kit, Thermo Scientific, Rockford, IL, USA) to normalize TG content following the manufacturer’s instructions.

The significance between control and treatment conditions was analyzed by one-way analysis of variance (ANOVA). Statistical comparisons of the different treatments were performed using Tukey’s test. All statistical analyses were performed using the GraphPad Prism 4 software (GraphPad Software, Suite, San Diego, CA, USA).

### 2.6. Oxidative Stress Assays in C. elegans

Populations of age-synchronized worms were obtained by isolating eggs from gravid adults and hatching the eggs in NGM plates (as control media) or NGM plates with the different doses of commercial carotenoids. In order to get a comparison of the antioxidant capacity provided by carotenoids respect to other well-recognized antioxidant compounds, vitamin C (0.1 µg/mL, Sigma-Aldrich, St. Louis, MO, USA) was used as positive control in the different oxidative stress assays. Experiments were performed according to a previously published protocol [57]. At least two independent experiments were performed with each sample.

Viability of worms between nematodes cultured in control and treatment conditions after oxidative stress were evaluated by means of one-way analysis of variance (ANOVA) and the Tukey test for comparative analysis. All the analyses were performed with the GraphPad Prism 4 statistical software (GraphPad Software, Suite, San Diego, CA, USA).

### 2.7. Transcriptomic Analysis in C. elegans

Gene expression in *C. elegans* N2 strain was analyzed in worm populations treated in NGM or NGM supplemented with BCX at 0.025 µg/mL dose. Synchronized populations were obtained from embryos isolated from gravid adults in the different feeding conditions. Worms were incubated at 20 °C. Then, 5-day adult worms were collected with M9 buffer, washed three times, and collected in 1.5-mL tubes for worm disruption by sonication (3 pulses at 10 W, 20 s/pulse). Total RNA isolation was performed with RNeasy kit (Qiagen, Barcelona, Spain). The RNA samples were processed for hybridization using the GeneChip^®^ *C. elegans* Genome Arrays of Affymetrix (UCIM, University of Valencia, Valencia, Spain). These chips contain oligonucleotide probe sets designed to asses over 22,500 transcripts from the *C. elegans* genome. Three biological replicates were examined per condition by bioinformatics. Raw data obtained from Affymetrix arrays were background corrected using the RMA (Robust Multi-Array Average) methodology [58]. Signal intensity was standardized across arrays via quantile normalization algorithm. Limma moderated *t*-statistics was used to evaluate differential gene expression between control and treated conditions. To control the false discovery rate, *p*-values were corrected for multiple testing as is described by Benjamini and Hochberg [59]. Finally, gene set analysis was performed for each comparison using logistic regression models [60].

## 3. Results

### 3.1. β-Cryptoxanthin Reduces Fat Content in C. elegans N2 in a Dose-Dependent Manner

**Figure 1:**
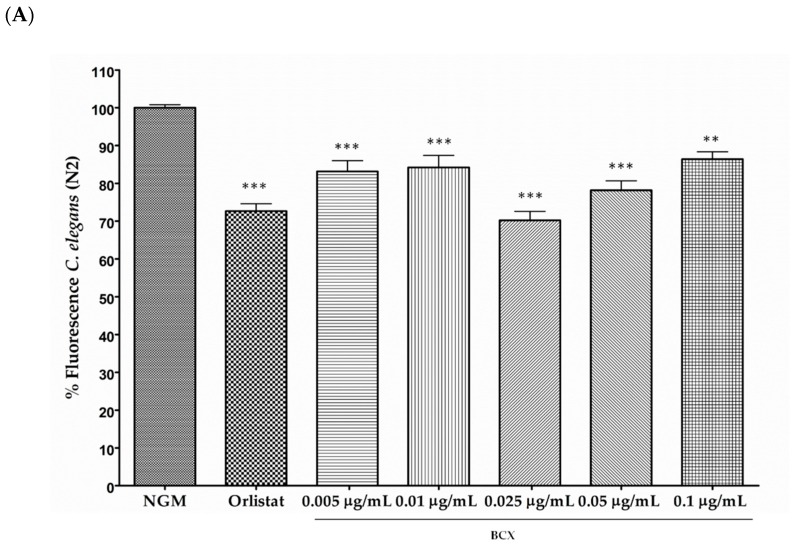

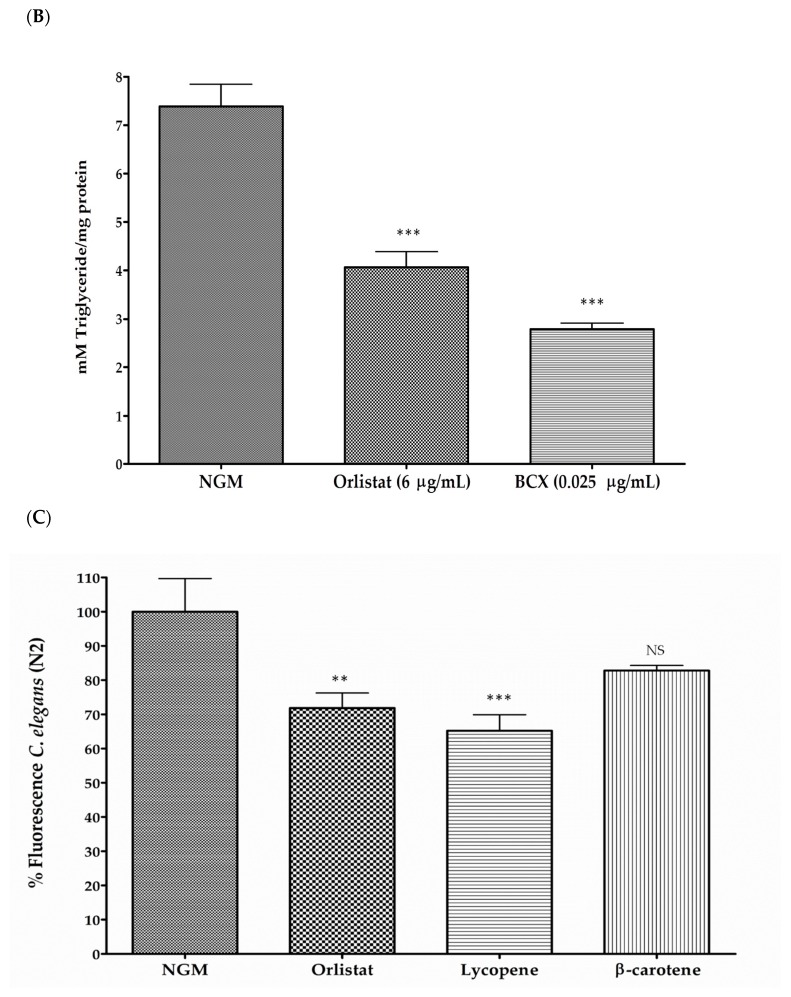
Body fat reduction in *Caenorhabditis elegans* provided by BCX (β-Cryptoxanthin) and other carotenoids. (**A**) Dose-response Nile Red assay with different doses of commercial β-cryptoxanthin. Nematode growth medium (NGM) was used as control feeding condition and Orlistat as positive control. Percentage of fluorescence is the mean of four independent experiments; (**B**) Quantification of triglycerides (TG) content (mM TG/mg protein) in *C. elegans* fed with BCX (0.025 µg/mL), Orlistat, and NGM; (**C**) *C. elegans* were fed with carotenoids lycopene and β-carotene at the dose of 0.025 µg/mL. Data are the average of at least three independent experiments. *** *p*-value ≤ 0.001, ** *p*-value ≤ 0.01, NS: not significant.

The effect on BCX on body-fat reduction, expressed as percentage of fluorescence reduction, in nematode populations fed with different concentration (0.005, 0.01, 0.025, 0.05, and 0.1 µg/mL) of the xanthophyll versus control feeding is shown in Figure 1A. All BCX doses assayed produced a significant body fat reduction, and ranged from 13.6% to 29.8% in respect to feeding under control conditions (*p* ≤ 0.001). However, the most effective dose was 0.025 µg/mL, with a fluorescence reduction of near 30%. These results indicate that BCX has a significant effect upon fat reduction in *C. elegans* in a dose-dependent manner.

Triglycerides (TGs) are the main constituents in lipid droplets stored in *C. elegans*. Therefore, TG quantification was performed with worms fed with BCX at the effective dose of 0.025 μg/mL to validate quantification of fluorescence in Nile Red stained worms. Results indicated a significant reduction in total TG in worms fed with BCX (*p* ≤ 0.001) compared with the control-fed nematodes (Figure 1B). Worms cultivated under control conditions presented a TG concentration of 7.39 mM/mg protein, while for BCX 0.025 μg/mL had a value of 2.79 mM/mg protein (a reduction of 62.23% of TG content in comparison to control conditions). This reduction was even higher than that of positive control Orlistat, with a TG content of 4.07 mM/mg protein (44.96% reduction over to control-fed nematodes). These results were consistent with the effect observed by Nile Red staining. Therefore, quantification of TG content in nematodes supports the reduction of total fat described in previous works [40,45].

To determine whether the fat reduction was a specific effect of BCX or may be also attributed to other carotenoids with health-related benefits, the fat reducing activity of lycopene and β-carotene was assessed in *C. elegans* (Figure 1C). Nematodes were fed with the concentration of 0.025 µg/mL of each carotenoid, since it was the most effective dose in body fat reduction in the BCX dose-response assays. Results showed a significant reduction in fluorescence produced by lycopene (34.8 %; *p* ≤ 0.01) whereas no significant effect was observed in nematodes fed with β-carotene (Figure 1C).

**Figure 2:**
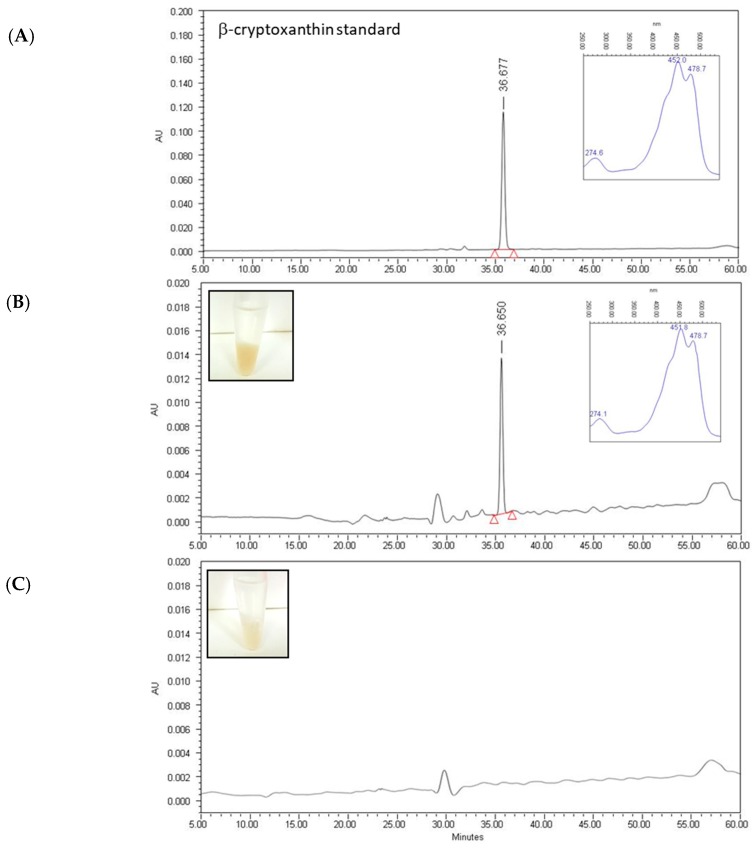
(**A**) BCX uptake by *C. elegans*. HPLC-PAD (High Performance Liquid Chromatography-Photodiode Array Detector) chromatograms of BCX (β-Cryptoxanthin) standard; (**B**) pellet extract from worms previously fed with BCX at 1 µg/mL; and (**C**) from worms grown under control condition (NGM (Nematode growth medium) without supplementation). Insert plots in (**A**,**B**) show the absorbance spectrum of the carotenoid peak identified in the chromatograms. Insert images in (**B**,**C**) show the corresponding worm pellets before disruption showing the differences in coloration. AU, arbitrary absorbance units.

To confirm the intake of BCX by the worms, experiments were performed by feeding nematodes with BCX and using the NGM without supplementation as a control. In these experiments we used a highest dose of BCX (1 µg/mL) to facilitate the detection of BCX in the worm extracts. The pellet corresponding to BCX-supplemented worms showed a cream-yellowish color while that of control populations showed a white-translucent color (Figure 2). The HPLC-PAD analysis showed the presence of BCX only in the extracts derived from worms fed with plates supplemented with BCX, whereas no carotenoids were detected in the extract of nematodes grown in control condition (Figure 2B,C). In the chromatogram from the worms extract grown in BCX supplemented media, a single peak with carotenoid spectrum was detected with a retention time and absorbance spectrum matching that of BCX standard (Figure 2A,B). This result indicates that BCX added to the culture media is uptake by the nematode.

**Figure 3:**
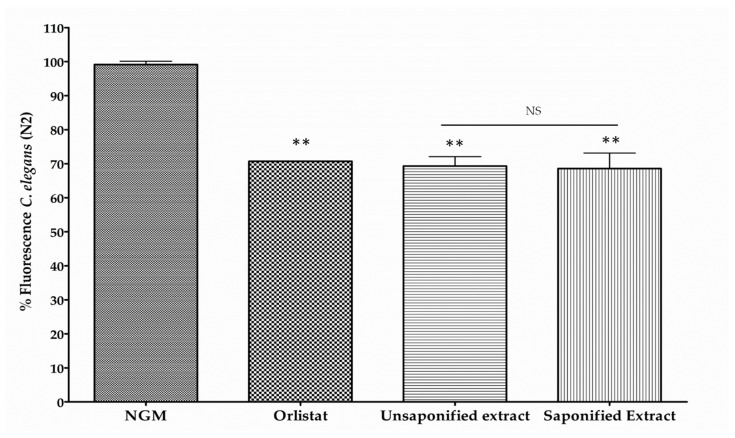
Measurement of fluorescence in Nile Red stained *C. elegans* worms fed with non-saponified or saponified carotenoid extracts from mandarin juice adjusted to contain 0.025 µg/mL of β-Cryptoxanthin (BCX). Nematode growth medium (NGM) was used as control feeding condition and Orlistat (6 µg/mL) as positive control. Percentage of fluorescence is the mean of two independent experiments. ** *p*-value ≤ 0.01, NS: not significant.

One important source of BCX in the human diet are mandarins which is esterified with a variety of fatty acids in a high proportion [8,12]. Analysis of carotenoid composition in the juice extract of Clementine mandarin showed that BCX was the main carotenoid accounting for nearly 60% of the total carotenoid content and being 90% in esterified form (Table 1). As expected, the mandarin extract also contained significant amounts of other β,β-xanthophylls (violaxanthin, zeaxanthin, and antheraxanthin), as well as β-carotene and colorless carotenes (phytoene and phytofluene) (Table 1). To investigate whether carotenoid esterification with fatty acids may have an impact on the reduction of lipid content in the nematode, the reduction in body fat was determined in nematodes fed with a non-saponified carotenoid extract from mandarin juice and compared with that of a saponified extract. Aliquots of free (saponified) or esterified (non-saponified) carotenoid extracts from mandarin juice were prepared to adjust the BCX to a final concentration of 0.025 µg/mL and the effect on worm body fat reduction was determined (Figure 3). Both types of extracts provide a significant reduction of fluorescence respect control medium (30.66% and 31.38%, respectively), without significant differences between them. Therefore, it appears that BCX in both free or esterified form provided a similar reduction in the body fat content of the nematodes.

**Figure 4:**
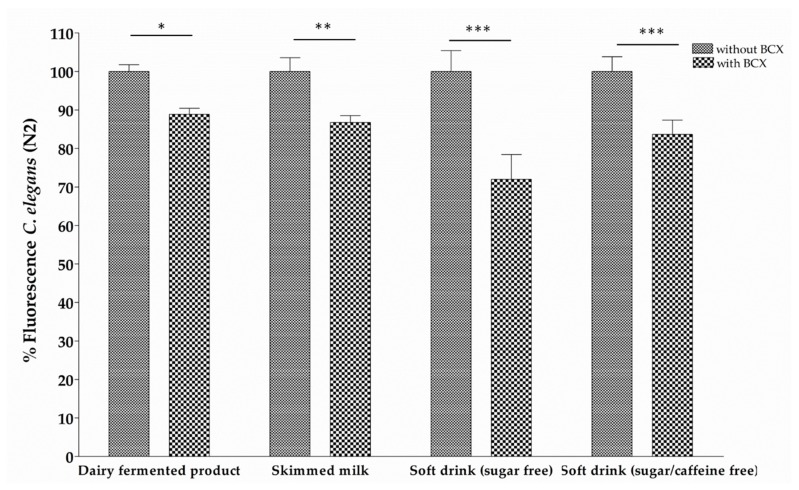
Body fat reduction measured by Red Nile staining in *C. elegans* worms fed with different food matrices supplemented with β-Cryptoxanthin (BCX) at 0.025 µg/mL. Nematode growth medium (NGM) was used as control feeding condition and Orlistat (6 µg/mL) as positive control. Percentage of fluorescence is the mean of two independent experiments. *** Significant *p*-value ≤ 0.001, ** Significant *p*-value ≤ 0.01, * Significant *p*-value ≤ 0.05.

To investigate the effect of different food matrices on the BCX-induced body fat reduction, a dairy fermented product, skimmed milk, and sugar free soft drink (with or without caffeine) were tested. Each food matrix was supplemented with the most effective BCX dose (0.025 µg/mL) and the lipid reduction was assayed in the nematode model for obesity. Figure 4 shows that all tested food matrices supplemented with BCX reduced body fat. However, sugar free soft drink with caffeine + BCX was the food matrix with a major fluorescence reduction (28.01%). These data indicate that addition of BCX to the different food matrices preserve its anti-obesity activity.

### 3.2. BCX has Antioxidant Activity in C. elegans N2

**Figure 5:**
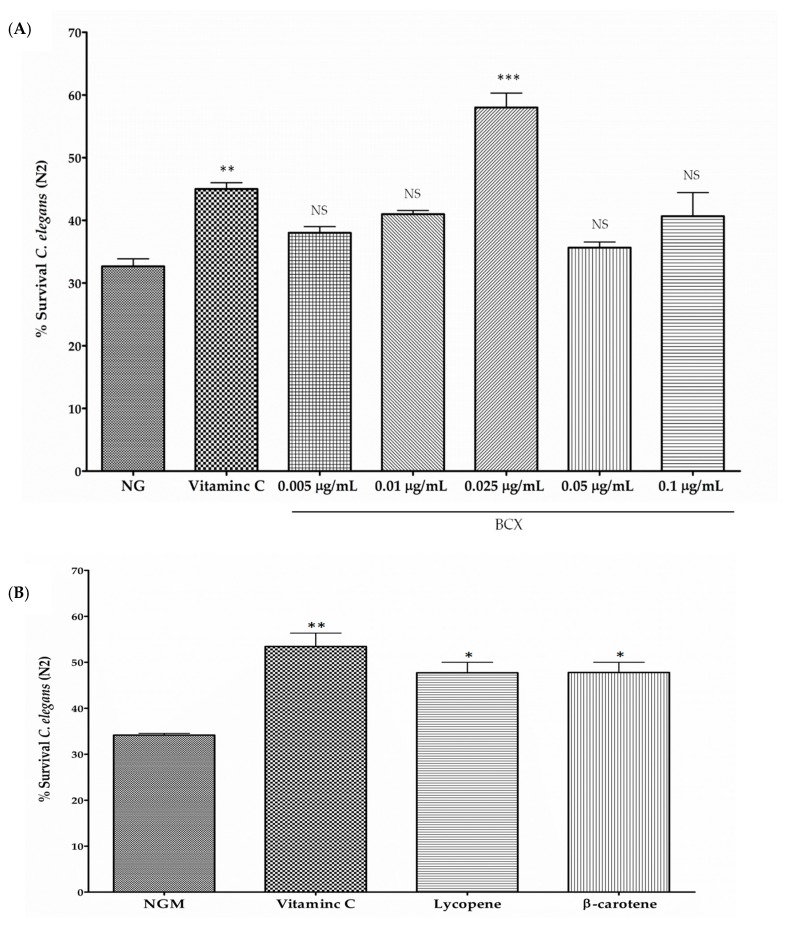
Percentage of *C. elegans* worm’s survival after an acute oxidative stress with 2 mM of H_2_O_2_. Vitamin C (0.1 µg/mL) was used as positive control. (**A**) Worms were fed at different concentrations of commercial β-Cryptoxanthin (BCX). Survival values correspond with the mean of three independent experiments; (**B**) Antioxidant activity of *C. elegans* fed with carotenoids lycopene and β-carotene at the dose of 0.025 µg/mL. In both experiments, Vitamin C (0.1 µg/mL) was used as antioxidant positive control. Survival values correspond with the mean of at least two independent experiments. *** *p*-value ≤ 0.001, ** *p*-value ≤ 0.01, * *p*-value ≤ 0.05, NS: not significant. NGM: nematode growth medium.

Carotenoids are recognized as antioxidants scavenging harmful reactive oxygen species (ROS) generated in the cell [14,61]. In order to check if BCX may also protect *C. elegans* N2 worms from oxidative stress, nematodes were fed with different doses of commercial BCX and subjected to an acute oxidative stress with H_2_O_2_ [57] (Figure 5A). Although the antioxidant capacity of carotenoids is different to that of the water-soluble molecules, an experiment with worms treated with ascorbic acid (0.1 µg/mL) was performed in order to include in the assays a well-recognized antioxidant as a positive internal control of the assay (not for comparative purposes). The survival percentage of H_2_O_2_-challenged nematodes was reduced to 33% whereas treatment with vitamin C (0.1 µg/mL) increased survival rate up to 45% (Figure 5A). Among the different BCX doses assayed, 0.025 µg/mL was the dose providing a significant increase in worm survival respect control medium, with survival percentage of 58%, that was even higher than that provided by vitamin C (Figure 5A). No significant effect on worm survival was observed with BCX concentrations lower or higher than 0.025 µg/mL. These results suggest that BCX exerts an antioxidant activity on nematodes after an acute oxidative stress at the same optimum concentration than that observed in fat reduction assays.

Furthermore, to compare the effect of BCX in respect to other carotenoids upon worms’ viability after an acute oxidative stress, we evaluated survival capacity in nematodes treated with lycopene and β-carotene at dose of 0.025 µg/mL, that showed the highest antioxidant effect for BCX (Figure 5A). Both carotenoids assayed produced a significant increase in nematode survival in respect to NGM medium in oxidative conditions, with very similar activity (48% of survival) (Figure 5B). Therefore, both carotenoids presented effective protection against an acute oxidative stress in nematodes, but to a lesser extent than BCX.

### 3.3. BCX Modulates Energy Metabolism, Antioxidant Response, and Protein Homeostasis in C. elegans

To understand the molecular mechanisms underlying the BCX effect in *C. elegans*, the transcriptomic profile of BCX-fed worms was studied using Affymetrix microarrays platform. Five-day adult worms were fed with BCX at 0.025 µg/mL, and the changes in gene expression as well as the study of functional blocks was made by comparing the transcriptomic profiling of BCX–treated worms versus control-fed worms.

First, differential expression at gene level was studied by analyzing the differences between BCX-treated samples and controls using limma moderated *t*-statistic. After feeding BCX, 542 genes were upregulated compared with control-fed nematodes. However, to get a stronger statistical significance, a *p*-value correction was applied, with no significant differences in gene expression. Therefore, a gene set analysis was performed by using two databases, GO (Gene Ontology) Biological Process and KEGG (Kyoto Encyclopedia of Genes and Genomes) Pathways, to facilitate the biological interpretation of microarray data. Thus, in BCX-treated worms, 18 metabolic pathways were found to be upregulated (*p* ≤ 0.05) compared with control nematodes (Table 2), while a total of 35 biological processes were upregulated (Table 3).

Regarding the metabolic pathways in BCX-treated worms, we found an upregulation of metabolic pathways related with energy metabolism (citrate cycle, pentose phosphate pathway, glycolysis/gluconeogenesis, and oxidative phosphorylation). Moreover, other pathways related to protein metabolism/processing were over-expressed under BCX supplementation (spliceosome, proteasome, and ubiquitin mediated proteolysis) (Table 2).

Concerning the non-redundant GO biological processes upregulated by BCX, different processes related to protein metabolism (translation, transport, and folding), as well as energy and carbohydrate metabolism processes (aerobic respiration, tricarboxylic acid cycle, glycolysis, oxidation of organic compounds), Notch signaling pathway and cell division were induced and cellular response to stress was also upregulated under BCX treatment (Table 3). All these processes were consistent with the observed upregulated KEGG pathways.

Finally, only three processes were found to be significantly downregulated in BCX-treated nematodes: potassium transport, sensory perception, and protein signaling pathway (Table 4).

## 4. Discussion

Xanthophyll β-cryptoxanthin is a xanthophyll with significant contribution to the pro-vitamin A activity in the human diet and its bio-accessibility from different food sources is similar or greater than that of α- or β-carotene [3,7]. The occurrence of BCX in food is variable and only a limited number of fruits and vegetables have moderate to high concentrations of BCX, being citrus fruits, especially mandarins and sweet oranges and their derivates, which are the major contributors to its intake in a common Western diet [6,9]. Besides the pro-vitamin A activity, in vitro, in vivo, and epidemiological studies suggest that BCX provides diverse health-related benefits, such as reducing risk of certain cancer [62], antioxidant [63], anti-inflammatory [15], anti-obesity [31], antidiabetic [33], and antiatherosclerosis properties [64]. Moreover, a positive effect on bone-related functions [23] and cognitive function [16] has been also described. However—in spite of the evidence of their beneficial properties in human nutrition and health—information about the mechanisms and metabolic pathways related to these beneficial effects of BCX are largely unknown [6].

**Figure 6:**
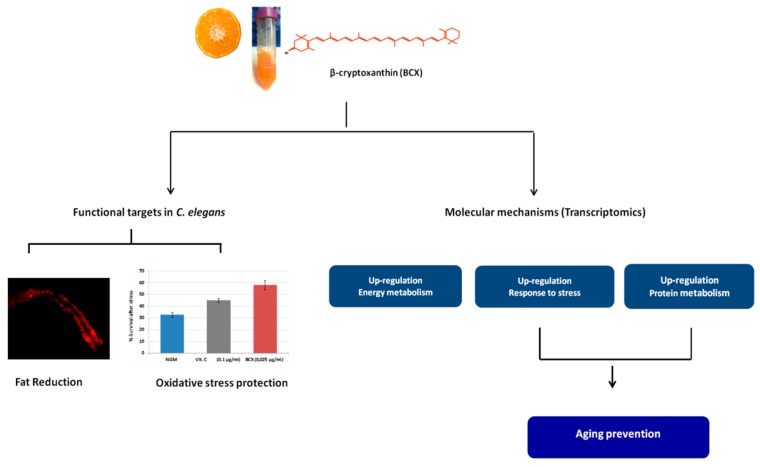
Model of the mechanism of action proposed for β-Cryptoxanthin (BCX) in *C. elegans*. Analysis of phenotype showed the efficacy of BCX on fat reduction and protection upon oxidative stress. The transcriptomic study provided the molecular mechanisms underlying these bioactivities, being the energy metabolism, the response to stress, and the protein metabolism the processes upregulated by BCX. NGM: nematode growth medium. VIt. C: Vitamin C.

*Caenorhabditis elegans* is a well-studied biological system and is considered an excellent model to study many health-related diseases, such as obesity and aging [37,65,66]. Moreover, other advantages of this model are that many of the pathways related to energy, lipid metabolism, and fat storage are conserved with humans [36]. Several researchers have used *C. elegans* to evaluate different ingredients with anti-obesity and antioxidant properties and to explore their mechanisms of action [40,42,45,67]. In the present study, we have used this nematode to investigate the anti-obesity and antioxidant effects of BCX (Figure 1 and Figure 5) and also to dissect the molecular responses caused by BCX intake in the nematode (Figure 6, Table 2 and Table 3). Our study demonstrates that BCX exhibits fat-reducing effect and protects nematodes from acute oxidative stress. Furthermore, BCX displays higher functional activity than other carotenoids as lycopene and β-carotene. This stronger effect of BCX has been previously postulated by other authors, specifically in relation with the improvement of inflammatory markers protecting against rheumatoid arthritis [20,68]. It can be speculated that the differential functional activity among carotenoids may be due to different bioassimilation or uptake. Analysis of carotenoids from worms fed with NGM media supplemented with BCX confirmed the BCX was uptake by the nematodes (Figure 2). The bioassimilation of other carotenoids by *C. elegans* was also demonstrated in feeding experiments with citrus pulp, where the presence of the β,β-xanthophyll violaxanthin, the main carotenoid in sweet orange pulp, was identified in worm pellets [47]. These results suggest that carotenoids are bioassimilated by *C. elegans* when added to the culture media either as a single ingredient or in a food matrix (Figure 2).

To understand the molecular mechanisms underlying the functional activity of BCX, a transcriptomic study was performed in *C. elegans*. Microarray analysis showed an upregulation of biological processes related to energy expenditure. Thus, citrate cycle (TCA cycle) and glycolysis were induced after BCX feeding. Taking into account that the major portion of glucose flux takes place through the TCA cycle [69], our results would suggest that BCX enhances glucose metabolism to ensure ATP demands for cells, being consistent with the anti-diabetic properties proposed for BCX [70]. Moreover, ATP-metabolic process such as oxidative phosphorylation pathway was upregulated in BCX-fed nematodes. Therefore, these findings would explain the fat-reduced phenotype observed in BCX-treated nematodes, and correlate with the mechanism of action exerted by other fat-reducing compounds, which induced the energy expenditure and oxidative stress response [45]. It is worthy to remark that the reduction of body fat accumulation observed in the nematode by supplementation of BCX was maintained in different food matrices (Figure 3). This observation is of particular practical interest since BCX would preserve its beneficial properties when added to different types of food preparation or supplementations, in accordance with the EFSA (European Food Safety Authority) recommendations. All these results, together with the previous evidence showing a reduction of visceral fat in mice, rats, and humans [29,31,71], reinforce the notion that BCX has an anti-obesity function in several biological systems.

The transcriptomic profile of BCX-treated nematodes also showed upregulation of processes related to cellular stress response. The observed transcriptomic response to oxidative stress produced by BCX was confirmed by survival analysis in nematodes treated with BCX and subjected to acute oxidative stress. Results indicate a marked antioxidant effect of BCX in a dose-dependent manner (Figure 5A), in agreement with other authors reporting the anti-inflammatory activity of BCX and its potential use for preventing vascular disease [20,68]. The antioxidant effect was even higher than that induced by other carotenoids with demonstrated antioxidant function as lycopene (Figure 5B) [72]. These results are consistent with those obtained with in vitro systems, where BCX also display potent scavenger activity against several ROS [73]. Under this system, BCX esterification with fatty acids did not affect scavenger activity compared to the free form and it was suggested that this modification may stabilize BCX to allow massive accumulation in plant biosynthetic tissues and also provides thermostability to the xanthophyll structure [11,73]. Moreover, a study in humans showed no differences in plasma response when BCX was given as esterified or non-esterified form, indicating a similar bioavailability of both [74]. This observation is also compatible with our results in the nematode model since no significant differences in the reduction of body fat were found between saponified or non-saponified carotenoid mandarin extracts where nearly 94% of the BCX was naturally accumulated in esterified form (Table 1). In other biological systems, antioxidant properties of BCX has also been observed, as the reduction of DNA oxidative damage in brain of aged mice [16], cultured Caco-2 and HeLa cells [75], or in the liver and the adipose tissues of rats [71]. Overall, these evidences suggest that BCX supplementation contributes to decrease cellular oxidative stress.

Obesity is thought to be a symptom of metabolic syndrome and leading to the production of oxidative stress and ROS [76]. Since our results show that BCX has anti-obesity and antioxidative effects in *C. elegans*, it is tempting to hypothesize that BCX may have a protective effect against development of the metabolic syndrome. Recent epidemiological studies indicate the relationship between the serum antioxidant status and the metabolic syndrome [32,34]. The metabolic syndrome is a complex disorder clustering obesity, diabetes mellitus, and atherosclerotic cardiovascular diseases [76]. It has been suggested that BCX supplementation is able to prevent metabolic syndrome in both mice and humans [28,29,31] and the BCX concentrations in serum are inversely related to indices of oxidative DNA damage and lipid peroxidation [33,77].

Analysis of the microarray data in nematodes fed with BCX also revealed an activation of several pathways related to protein metabolism, such as proteasome, translation, spliceosome, ubiquitin mediated proteolysis, and protein folding. A protein homeostasis process is associated with aging and neurodegeneration [78,79]. A failure in this homeostasis can result in protein misfolding and in the accumulation of insoluble protein fibrils and aggregates, such as amyloids. Thus, the search for compounds or molecules such as BCX that enhance protein turnover could have a beneficial effect against aging and age-related diseases. This suggestion is in accordance with previous report where mice fed with BCX showed a reduction of the risk of age-related cognitive dysfunction and higher learning ability [16].

In summary, the in vivo anti-obesity and antioxidant activity of BCX in the nematode model system has been described in this study. Our results suggest a molecular mechanism based on promoting energy expenditure and cellular response to stress pathways. Moreover, BCX also displays upregulation of biological processes related to protein homeostasis. Therefore, this study provides new evidences for the potential therapeutic use of BCX in the prevention of diseases related to metabolic syndrome and aging. In this respect, further studies would be necessary to confirm the role of BCX on aging-related diseases.

## Figures and Tables

**Table 1:** Carotenoid content and composition in saponified and non-saponified juice extracts of Clementine mandarins. The percentage of β-Cryptoxanthin over the total amount of carotenoids is indicated in parenthesis.

Carotenoid	Concentration (µg/mL) in Mandarin Juice Extract ^1^	
	Saponified	Non-saponified
Phytoene	0.80 ± 0.05	0.86 ± 0.03
Phytofluene	0.37 ± 0.09	0.45 ± 0.02
ζ-Carotene	0.19 ± 0.04	0.25 ± 0.03
β-Carotene	0.28 ± 0.05	0.35 ± 0.02
β-Cryptoxanthin	4.86 ± 0.23 (61%)	0.30 ± 0.02 (3%)
Zeaxanthin	0.11 ± 0.02	N.D.
Anteraxanthin	0.52 ± 0.01	N.D.
Violaxanthin ^2^	0.75 ± 0.01	N.D.
**Esters (mono- and diesters)**		
β-Crytoxanthin	N.D.	5.35 ± 0.25 (57%)
Zeaxanthin	N.D.	0.20 ± 0.01
Antherxanthin	N.D.	0.70 ± 0.03
Violaxanthin ^2^	N.D.	0.92 ± 0.04
Total carotenoids	7.88 ± 0.62	9.38 ± 0.48

^1^ Data are mean ± SD (*n* = 3); ^2^ Sum of 9-*Z*- and *all*-*E*-isomers; N.D. Not detected.

**Table 2:** KEGG (metabolic pathways were significantly (*p* ≤ 0.05) upregulated in *C. elegans* N2 after feeding with β-Cryptoxanthin (BCX) (0.025 µg/mL) as compared with control feeding conditions.

Upregulated KEGGSs Pathways (BCX vs. Control)			
ID	Name	Size ^a^	*p*-Value
04141	Protein processing in endoplasmic reticulum	170	1.26 × 10^9^
00020	Citrate cycle (TCA cycle)	42	0.0005
03040	Spliceosome	123	0.0025
00010	Glycolysis/gluconeogenesis	38	0.0067
03030	DNA replication	39	0.0088
00970	Aminoacyl-tRNA biosynthesis	49	0.0118
03050	Proteasome	45	0.0180
00030	Pentose phosphate pathway	26	0.0180
04144	Endocytosis	109	0.0231
00520	Amino sugar and nucleotide sugar metabolism	32	0.0232
04130	SNARE interactions in vesicular transport	23	0.0232
00240	Pyrimidine metabolism	71	0.0232
00052	Galactose metabolism	13	0.0247
00051	Fructose and mannose metabolism	31	0.0247
00564	Glycerophospholipid metabolism	54	0.0323
04330	Notch signaling pathway	28	0.0393
00190	Oxidative phosphorylation	127	0.0393
04120	Ubiquitin mediated proteolysis	111	0.0393

^a^ Number of genes among the group. (KEGG: Kyoto Encyclopedia of Genes and Genomes; TCA: Tricarboxylic Acid Cycle); SNARE: Soluble NSF Attachment Protein Receptor.

**Table 3:** GO Biological Processes was significantly (*p* ≤ 0.05) non-redundantly upregulated in *C. elegans* N2 feeding with β-Cryptoxanthin (BCX) (0.025 µg/mL) as compared with control feeding conditions.

Non-Redundant Upregulated GOs (BCX vs. Control)	
ID	Name
0000910	Cytokinesis
0048598	Embryonic morphogenesis
0006886	Intracellular protein transport
0006400	tRNA modification
0006479	Protein amino acid methylation
0006099	Tricarboxylic acid cycle
0006412	Translation
0019751	Polyol metabolic process
0006261	DNA-dependent DNA replication
0006457	Protein folding
0007346	Regulation of mitotic cell cycle
0030261	Chromosome condensation
0008593	Regulation of Notch signaling pathway
0008284	Positive regulation of cell proliferation
0040028	Regulation of vulval development
0000075	Cell cycle checkpoint
0001708	Cell fate specification
0040019	Positive regulation of embryonic development
0007067	Mitosis
0008356	Asymmetric cell division
0006096	Glycolysis
0040015	Negative regulation of multicellular organism growth
0007283	Spermatogenesis
0051246	Regulation of protein metabolic process
0040039	Inductive cell migration
0033554	Cellular response to stress
0000132	Stablishment of mitotic spindle orientation
0045132	Meiotic chromosome segregation
0045137	Development of primary sexual characteristics
0035194	Posttranscriptional gene silencing by RNA
0007143	Female meiosis
0046580	Negative regulation of Ras protein signal transduction
0000184	Nuclear-transcribed mRNA catabolic process, nonsense-mediated decay
0022613	Ribonucleoprotein complex biogenesis
0051302	Regulation of cell division

GO: Gene Ontology.

**Table 4:** GO Biological Processes was significantly (*p* ≤ 0.05) non-redundantly downregulated in *C. elegans* N2 feeding with β-Cryptoxanthin (BCX) (0.025 µg/mL) as compared with control feeding conditions.

Non-Redundant Downregulated GOs (BCX vs. Control)	
ID	Name
0006813	Potassium ion transport
0007606	Sensory perception of chemical stimulus
0007186	G-protein coupled receptor protein signaling pathway

GO: Gene Ontology.
